# Microbial colonization patterns and biodegradation of petrochemical and biodegradable plastics in lake waters: insights from a field experiment

**DOI:** 10.3389/fmicb.2023.1290441

**Published:** 2023-12-06

**Authors:** Francesca Di Pippo, Valerio Bocci, Stefano Amalfitano, Simona Crognale, Caterina Levantesi, Loris Pietrelli, Valerio Di Lisio, Andrea Martinelli, Simona Rossetti

**Affiliations:** ^1^Water Research Institute, CNR-IRSA, National Research Council, Rome, Italy; ^2^PhD Program in Evolutionary Biology and Ecology, Department of Biology, University of Rome “Tor Vergata”, Rome, Italy; ^3^National Biodiversity Future Center, Palermo, Italy; ^4^DAFNE Department, Tuscia University, Viterbo, Italy; ^5^Donostia International Physics Center, Paseo Manuel de Lardizabal, San Sebastián, Spain; ^6^Department of Chemistry, Sapienza University of Rome, Rome, Italy

**Keywords:** plastisphere, plastics, freshwater, biofilms, biodegradation

## Abstract

**Introduction:**

Once dispersed in water, plastic materials become promptly colonized by biofilm-forming microorganisms, commonly known as plastisphere.

**Methods:**

By combining DNA sequencing and Confocal Laser Scanning Microscopy (CLSM), we investigated the plastisphere colonization patterns following exposure to natural lake waters (up to 77 days) of either petrochemical or biodegradable plastic materials (low density polyethylene - LDPE, polyethylene terephthalate - PET, polylactic acid - PLA, and the starch-based MaterBi® - Mb) in comparison to planktonic community composition. Chemical composition, water wettability, and morphology of plastic surfaces were evaluated, through Transform Infrared Spectroscopy (ATR-FTIR), Scanning Electron Microscopy (SEM), and static contact angle analysis, to assess the possible effects of microbial colonization and biodegradation activity.

**Results and Discussion:**

The phylogenetic composition of plastisphere and planktonic communities was notably different. Pioneering microbial colonisers, likely selected from lake waters, were found associated with all plastic materials, along with a core of more than 30 abundant bacterial families associated with all polymers. The different plastic materials, either derived from petrochemical hydrocarbons (i.e., LDPE and PET) or biodegradable (PLA and Mb), were used by opportunistic aquatic microorganisms as adhesion surfaces rather than carbon sources. The Mb-associated microorganisms (i.e. mostly members of the family Burkholderiaceae) were likely able to degrade the starch residues on the polymer surfaces, although the Mb matrix maintained its original chemical structure and morphology. Overall, our findings provide insights into the complex interactions between aquatic microorganisms and plastic materials found in lake waters, highlighting the importance of understanding the plastisphere dynamics to better manage the fate of plastic debris in the environment.

## 1 Introduction

The ubiquitous accumulation and slow degradation of plastic polymers in aquatic environment are posing an ever increasing ecological threat (Wang et al., 2021; Zhao et al., 2022; Wang et al., 2023). Once in water, larger plastic debris progressively breaks down into micro- and nano-plastics, which detrimentally affect the structural and functional traits of aquatic biota and the related ecosystem functions (Khalid et al., 2021; Zhang T. et al., 2022; Adegoke et al., 2023).

Recent studies recognized the unique role of the emerging plastic pollution in providing a novel niche suitable for typical benthic organisms in pelagic environment, with largely unexplored ecological implications (Yang et al., 2020; Dąbrowska, 2021; Haram et al., 2021; Li et al., 2021). Plastics and micro-plastics (MPs) can be vectors of chemical and microbiological contaminants, along with biofilm-forming microorganisms (Di Pippo et al., 2022; Du et al., 2022; Rubin and Zucker, 2022). The complex plastic-associated microbiota, referred to as the plastisphere (Zettler et al., 2013), is composed by primary producers, grazers, predators, and decomposers with biodiversity profiles consistently different from those of surrounding waters (Odobel et al., 2021; Du et al., 2022; Li et al., 2023; Nikolopoulou et al., 2023). A large body of the recent literature was dedicated to characterize the species composition of marine plastisphere, while relatively less studies focused on the plastisphere in freshwater ecosystems (McCormick et al., 2014, 2016; Hoellein et al., 2017; Di Pippo et al., 2020, 2022). Several environmental conditions and local factors, including seasonal variability, biogeographical patterns, and anthropogenic impact were reported to influence microbial communities developing on plastic surfaces (Hoellein et al., 2017; Oberbeckmann et al., 2018; Amaral-Zettler et al., 2020; Coons et al., 2021; Amaneesh et al., 2023). However, the role of polymer types and properties contributing to drive plastisphere structure and biodiversity is still unclear (Jacquin et al., 2019; Coons et al., 2021; Sooriyakumar et al., 2022; Wang et al., 2022; Li et al., 2023; Miao et al., 2023). Plastisphere diversity was found to respond to polymer properties (Oberbeckmann et al., 2016; Kettner et al., 2019), but the extent to which taxa composition is affected by plastic type over time is not fully understood. The attachment of microorganisms in the early developmental stages and the microbial biofilm maturation are influenced by chemical, physical, mechanical and morphological properties of polymer substrata (Dexter, 1979; Kreve and Reis, 2021; Zheng et al., 2021; Jia, 2022). Additionally, the presence of plastic additives or potential organic contaminants can lead to structural variations, thus altering the original properties of the bare solid surface (Bhagwat et al., 2021).

Most of the available studies analyzed biofilms on plastics and MPs in environmental samples, with limited information regarding exposure times and persistence in the aquatic environment. Therefore, it is not possible to discriminate the effect of the polymer type from that of microbial biofilm development. Moreover, given the few field studies investigating microbial colonization of plastic items *in situ*, a consistent knowledge gap exists regarding freshwater plastisphere development and establishment patterns over time and on microbial taxa involved in the different stages of community succession (Amaral-Zettler et al., 2020; Eronen-Rasimus et al., 2022; Forero-López et al., 2022; Wallbank et al., 2022; Nguyen et al., 2023). A deeper knowledge on plastic-associated biofilm communities is critical to understand the role of plastisphere in plastic biodegradation (Jacquin et al., 2019; Eronen-Rasimus et al., 2022). Current data rely mainly on culture-based approaches with selected strain, under optimal laboratory conditions, which do not reflect the natural ones (Mierzwa-Hersztek et al., 2019; Mohanan et al., 2020; Amobonye et al., 2021; Nguyen et al., 2021). Only few on-site studies have been performed in freshwaters (Nguyen et al., 2023). Further, the adopted standards and test methods are insufficient to realistically predict the plastic biodegradability in aquatic environments, owing to several shortcomings in experimental procedures (Harrison et al., 2018; Ferreira-Filipe et al., 2022).

The degradation of plastic items can involve changes in bulk and surface material structure (e.g., holes, cracks, weight loss), along with alterations in chemical composition, morphology, roughness, thermal, and mechanical properties (Chen et al., 2020; Denaro et al., 2020; Zurier and Goddard, 2021; Kosiorowska et al., 2022; Li et al., 2023). Unfolding the plastisphere colonization patterns and understanding their possible relations with polymer modifications can provide novel insights on the fate of these materials in natural aquatic environments.

This study was entailed at investigating the effects of polymer type on freshwater plastisphere microbial community composition and diversity over time. More specifically, we pursued the following goals: (i) to explore the lake plastisphere diversity at varying plastic types and immersion times during biofilm formation; (ii) to assess whether pioneering microorganisms that promptly colonize plastics are recruited from the freshwater source community; (iii) to identify the occurrence of a core microbial community and plastic-degrading taxa emerging during biofilm development as well as plastic possible deterioration.

We also investigated how plastisphere patterns could affect the chemical and morphological modifications of the polymer surface structure, with direct consequences on the integrity of plastic samples and their persistence in freshwater environments.

An on-site exposure experiment was performed at Lake Bracciano (Italy) by targeting the freshwater microbial biofilms grown onto fragments of different plastic types, including low density polyethylene (LDPE), polyethylene terephthalate (PET), polylactic acid (PLA), and the starch-based bioplastic MaterBi^®^ (Mb).

## 2 Materials and methods

### 2.1 Experimental design and field work

The experiment was carried out in a selected coastal site of Lake Bracciano (42° 7′ 10.292″ N 12° 14′ 36.052″ E; 164 m a.s.l.; Latium, Italy). With a surface area of 51 km^2^, a maximum depth of 165 m, and a water volume of 5.5 km^3^, the lake represents a fundamental source of quality freshwaters in Central Italy and a popular touristic destination spot. Recent investigations revealed the occurrence of plastic waste and abundant micro-plastics in the lake waters (Corti et al., 2020; Cera et al., 2022; Di Pippo et al., 2022).

We selected plastic polymer coupons specifically taken from daily-life plastic products, including low density polyethylene (LDPE) from a shopping bag, polyethylene terephthalate (PET) from a water bottle, polylactic acid (PLA) from a disposable dish, and the starch-based bioplastic MaterBi^®^ (Mb) from a compostable shopping bag.

For each polymer type, 20 coupons of fixed size (approx. 2×3 cm) were prepared and rinsed with 70% ethanol solution for surface cleaning (Syranidou et al., 2019). The coupons were placed in lake waters by fixing them to a nylon fishing line (stretched between two wooden stakes of a jetty) and kept submerged at about 50 cm under the water surface. Samples were collected after 7 (t1), 17 (t2), 38 (t3) and 77 (t4) days of field incubation. The immersion time period was specifically in agreement with the incubation time of standard tests for polymer biodegradability (Science Advice for Policy by European Academies [SAPEA], 2020). For each type of plastic, additional reference polymer coupons were preserved for control analysis (t0). The sample code was Xtn, where X is the polymer acronym (LDPE, PET, PLA, and Mb) and tn the exposure time (t0, t1, t2, t3 and t4). Two collected coupons for each sampling time were gently washed with sterile saline solution (0.9%) to rinse off non-attached organisms, fixed (formaldehyde = 5% final concentration), and stored at −20°C until CARD-FISH analyses. The remaining replicates were washed with sterile saline solution (0.9%) and stored at −20°C until further DNA extractions (Di Pippo et al., 2022).

At each sampling event, two samples of lake water (2 × 500 mL) were collected in sterile bottles and directly filtered on polycarbonate membranes (pore size 0.2 mm, 47 mm diameter, Nuclepore) by gentle vacuum (<0.2 bar). Filters were stored at −20°C until analyses (Supplementary Figure 1).

### 2.2 CAtalyzed reporter deposition fluorescence *in situ* hybridisation (CARD-FISH) and confocal laser scanning microscopy (CLSM)

Plastic coupons, two for each polymer and sampling time, were fixed (formaldehyde = 5% final concentration) for CLSM observation and stored at −20°C. CARD-FISH, with horseradish peroxidase labeled oligonucleotide probes (Biomers, Ulm, Germany) and signal amplification with fluorescein labeled tyramides, was performed as described in Matturro et al. (2012). CARD-FISH hybridization EUB338mix probes and DAPI staining (1.5 μg mL^−1^, Vector Labs, Milano, Italy) were used to identify bacteria and total microorganisms in biofilm, respectively. Photosynthetic prokaryotic and eukaryotic cells were visualized due to their constitutive content of auto-fluorescent pigments. Biofilm samples were observed by CLSM using a FV1000 (Olympus Corp., Tokyo, Japan) in multichannel mode (Di Pippo et al., 2020) for visualization of microbial cell clusters grown onto surfaces of all plastic types and at all sampling times (different biofilm development stages). 3-D images were re-constructed from 2-D cross-sectional images (x–y plane; 0.5-μm intervals) and 3-D volume renderings in blend mode were generated in maximum intensity projection (MIP) by using Imaris 6.2.0 software (Bitplane AG, Zurich, Switzerland).

### 2.3 DNA extraction from surface-attached and planktonic communities

DNA from plastic and water samples was extracted by using DNeasy PowerSoil Pro Kit (QIAGEN - Germantown, MD) following manufacturer’s instructions. Purified DNA from each sample, eluted in 100 μL sterile Milli-Q water, was stored at−20°C. Nanodrop 3300 (Thermo Scientific, Italy) was used to assess the quality of extracted DNA (1.6 < A260/280 < 1.8 and A260/230 > 2). The DNA extracted from the negative controls were negative, thus they were not further processed.

Additional tests were performed by SEM and ATR-FTIR to exclude possible effects of DNA extraction procedure on morphology and composition of virgin polymer samples (Supplementary materials).

### 2.4 Quantitative PCR (qPCR)

qPCR was used to quantify total microbial load by 16S rRNA gene quantification (Suzuki et al., 2000) in DNA extracted from water and plastic samples experimental replicates. qPCR was performed with CFX96^™^ Real-Time PCR Detection System (Bio-Rad Laboratories, Hercules, CA) in 96 well plates. Each 20 μL volume reaction contained 3 μL of DNA template (3–5 ng/ μL), 10 μL of 2X SYBR Green Supermix (Bio-Rad USA), and finally primers (10 μM) for optimized Q-PCR amplification (Di Pippo et al., 2022). The amount of target genes in unknown samples was calculated based on a standard curve (Ct value versus log of initial gene copy number) obtained using serial dilutions of a positive control. Data were analyzed with the CFX Manager^™^ software (version 3.1, Bio-Rad, Italy). The 16S rRNA genes, used as positive control was produced by PCR amplification, quantified with NanoDrop spectrophotometer and finally the gene copy number per μL of genomic DNA solution was calculated as described by Czekalski et al. (2012). Ten-fold serial dilutions of genomic DNA (gene copy numbers from 10^3^ to 10^9^ per reaction tube) were amplified with unknown samples and no template control in triplicate for each qPCR run. Results were reported as the average of measurements with standard deviations and the amount of 16S rDNA was assessed after normalization to plastic surface (gene copy number/plastic cm^−2^).

### 2.5 16S rRNA gene sequencing and bioinformatics

Extracted DNA was amplified in a first PCR with the primer pair 27F (5′-AGAGTTTGATCCTGGCTCAG-3′) and 534R (5′-ATTACCGCGGCTGCTGG-3′), targeting the V1-V3 region of bacterial 16S rRNA gene. Reactions were set up in 25 μL volumes containing 15 ng of DNA, 0.5 μM primers and 1× Phusion High-Fidelity PCR Master Mix (Thermo Fisher Scientific, Waltham, MA USA). PCR settings: initial denaturation at 98°C for 10 s, 8 cycles of 98°C for 1 s, 55°C for 5 s, 72°C for 15 s and final elongation at 72°C for 1 min. Amplicon libraries were purified using the Agencourt^®^ AMpure XP bead protocol (Beckmann Coulter, USA). Library concentration was measured with Qubit 3.0 Fluorometer (Thermo Fisher Scientific, Waltham, MA USA). Purified libraries were pooled in equimolar concentrations and diluted to 4 nM. The samples were paired and sequenced (2×301 bp) on a MiSeq platform (Illumina) using a MiSeq Reagent kit v3, 600 cycles (Illumina, USA) following standard guidelines for preparing and loading samples. 10% Phix control was used in the pooled libraries to overcome low complexity issue often observed with amplicon samples.

After checking read quality with fastQC, sequences were processed and analyzed using QIIME2 software tools (2018.2 release version). Reads were demultiplexed using demux plugin^1^ and the primer sequences were removed by using Cutadapt plugin.^2^ Demultiplexed reads were denoised, dereplicated and chimera-filtered and amplicon sequence variants (ASVs) were obtained using the DADA2 package (Callahan et al., 2016). According to Campbell et al. (2011), abundant ASVs were defined as those that comprised 1% or more of the community. Dominant ASVs were herein defined as those showing a relative abundance >10% of the total reads.

The raw 16S rRNA gene sequences are available through the Sequence Read Archive (SRA) under accession number PRJNA905066.

### 2.6 ATR-FTIR measurements

Following DNA extraction, the plastic coupons were rinsed with 2% SDS, water and further analyzed for evaluating possible changes of: (i) surface chemical structures, (ii) hydrophobicity, and (iii) morphology after plastisphere colonization. The results were compared with those obtained from reference polymer coupons. Attenuated total reflection Fourier transform infrared analysis (ATR-FTIR) was performed using a Thermo Nicolet 6700 FT-IR Spectrometer equipped with a Specac Golden Gate diamond single reflection accessory. Spectra were acquired on both sides of the films in the range 4,000–650 cm^−1^ by co-adding 100 scans with a resolution of 2 cm^−1^.

### 2.7 Scanning Electron Microscopy (SEM)

The morphology of the plastic coupon surfaces (reference polymer and plastic at t4 sampling time) was investigated by Scanning Electron Microscopy (SEM, AURIGA, Zeiss, Jena, Germany). Prior to the measurements, the samples were dried under vacuum at room temperature and then gold-sputtered.

### 2.8 Static contact angle

Contact angle analysis, commonly applied to assess polymers surface energy, provided valuable information on the overall polymer wettability. The data are dependent on surface modifications (i.e., chemical composition, roughness, heterogeneity), which provide an indication of possible abiotic or biotic deterioration activities. Static contact angle measurements were performed by dropping 2 μL of Milli-Q water on the vacuum-dried polymer samples. Images were successively acquired by a home-made system that allowed translating the sample along the vertical and horizontal axis. Static contact angle was calculated by using the Motic Image Plus 2.0 software (Motic China Group Co.). The contact angles are the average of measurements carried out on three replicate analyses (three drops placed on three samples) at all the different immersion periods.

### 2.9 Statistical analyses

The univariate non-parametric Kruskal–Wallis test for equal medians, along with the Mann–Whitney pairwise comparison, was performed to assess differences in microbial load, using the log-transformed qPCR data, across sampling times (i.e., t1 vs. t2 vs. t3 vs. t4) and polymer types (i.e., LDPE vs. PET vs. PLA vs. Mb). Non-parametric multivariate analysis of variance (PERMANOVA) was performed to test the differences in microbial community composition between environmental matrices (water vs. plastisphere), sampling times (i.e., t1 vs. t2 vs. t3 vs. t4), and polymer types (i.e., LDPE vs. PET vs. PLA vs, Mb). Similarity matrices of bacterial community composition were calculated using sequencing data and applying the relative abundance-based Bray-Curtis index at the family level. A Non-metric Multi-Dimensional Scaling analysis (NMDS) was used to visualize the variation patterns of water and plastisphere bacterial communities. Relative abundance values of dominant bacterial families in the plastisphere (>10% of total reads) were incorporated into the analysis with a vector-fitting procedure, in which the vector length is proportional to the correlation coefficient between each variable and the NMDS axis scores. Detrended Correspondence Analysis (DCA) was applied to the phylogenetic dataset to identify the distribution of abundant bacterial families (>1% of total reads) recovered from all polymer types, allowing for the identification of core taxa in the plastisphere. All statistical analyses were performed by PAST software package (PAlaeontological STatistics, ver. 4.04).

## 3 Results

### 3.1 Detection of biofilm-forming microorganisms and community structure

All plastic coupons showed biofilm growth over the experimental time, and the CLSM examination showed the co-occurrence of heterotrophic and photosynthetic pigmented cells in all plastispheres.

**Figure 1:**
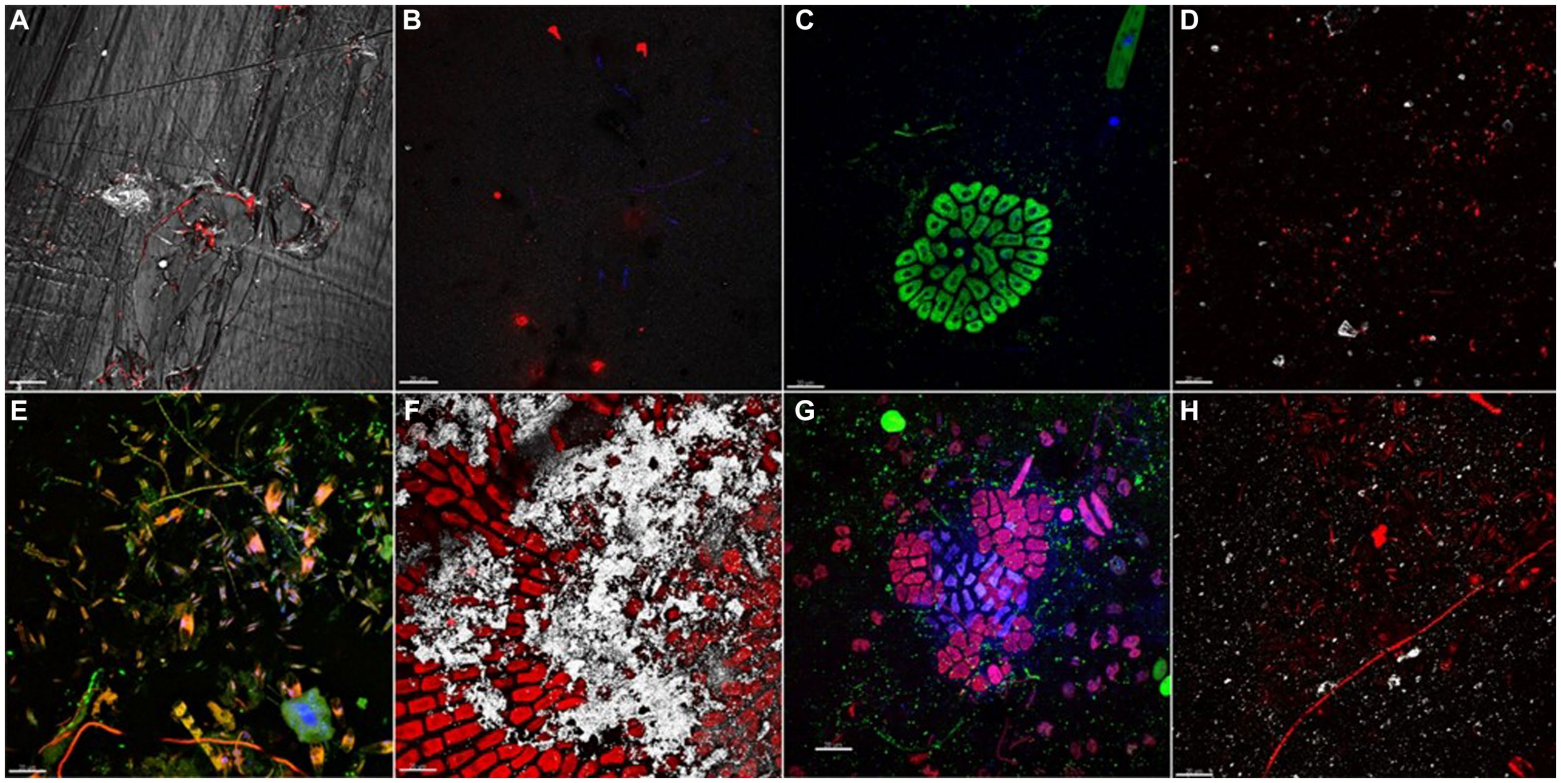
Biofilm communities, grown on different plastic types after 7 **(A–D)** and 77 **(E–H)** days of immersion. **(A)** PET plastisphere: bacteria targeted with probe EUB338mix (red signal) merged with PET plastic surface acquired in reflection mode (white signal). **(B)** Bacteria targeted with probe EUB338mix (red signal) and total viable cells stained with DAPI (blue signal) in LDPE plastisphere. **(C)** Mb plastisphere: total viable cells stained with DAPI (blue signal) autofluorescence of chlorophyll *a* (green signal). **(D)** PLA plastisphere: bacteria targeted with probe EUB338mix (red signal) and plastic surface acquired in reflection mode (white signal). **(E)** PET plastisphere: bacteria targeted with probe EUB338mix (green signal) and autofluorescence of chloropyll *a* of diatoms chloroplasts and of filamentous cyanobacteria (red/orange signal) merged with total viable cells stained with DAPI (blue signal). **(F)** LDPE plastisphere: autofluorescence of chloropyll *a* (red signal) and LDPE plastic surface (white signal). **(G)** Mb plastisphere: bacteria targeted by probe EUB338mix (green signal), autofluorescence of green algae chloropyll *a* (red signal) merged with biofilm total viable cells stained with DAPI (blue signal). **(H)** PLA plastisphere: bacteria hybridized with EUB338mix probe (red signal) and PLA plastic surfaces (white signal). The scale bar was set at 20 μm.

At initial stage of development (t1, day 7), different distribution of bacterial cells was observed onto plastic surfaces (Figures 1A–D). LDPE, PET, and PLA were mainly colonized by bacterial cells heterogeneously distributed on coupon surfaces, with no evident differences between replicates of same material. Mb biofilm showed a diverse composition, comprising bacterial micro-colonies and patchy distributed pigmented eukaryotes (i.e., green algae and pennate diatoms). All biofilms, collected at t4 (day 77), showed increased levels of plastisphere complexity in respect to t1. CLSM examination showed diverse taxa of pennate diatoms, colonial/filamentous green algae, and non-photosynthetic bacterial clusters closely associated with cyanobacterial filaments (Figures 1E–H). The 3-D reconstruction of the biofilm topology showed a highly complex and multi-stratified structure of plastisphere in all polymer coupons (Supplementary Figure 2).

### 3.2 Bacterial community composition across polymer types and water immersion times

As assessed by qPCR for the 16S rRNA gene quantification, the microbial load changed significantly over time and between polymers (Kruskal–Wallis test, *p* < 0.001). Average values increased significantly from t1 (mean = 3.62 × 10^6^ gene copies cm^−2^) to t3 (mean = 2.19 × 10^7^ gene copies cm^−2^). The lowest values were found in Mb (mean = 8.01 × 10^5^ gene copies cm^−2^) (Supplementary Table 1).

The analyses of 16S rRNA gene sequences showed similar values of alpha-diversity of plastisphere communities over time and regardless the polymer types (Supplementary Table 2). The rank-abundance curves showed few abundant ASVs (>1% total reads) and a long tail of rare ASVs (<1% total reads) (Supplementary Figure 3). Although the abundant ASVs were affiliated to only 5.1% of the total taxa on average, they contributed to most of the retrieved sequences (50.5% of the total reads). Moreover, significant differences in the phylogenetic composition between water and plastisphere communities (PERMANOVA, *p* < 0.001) (Supplementary Figure 4). The phylum Actinobacteria (33.3–66.4% of total reads) dominated the planktonic community, mainly comprising the classes Actinobacteria (up to 53.2%) and Acidimicrobiia (up to 19.3%), represented by families Microbacteriaceae (class Actinobacteria), Ilumatobacteraceae (class Acidimicrobiia), and Sporichthyaceae (class Actinomycetia) (Supplementary Figure 5). Members of class Alphaproteobacteria (13.6–27.7% of total reads) were also abundant and mainly represented by Sphingomonadaceae (up to 6.5%) (Supplementary Figure 5).

Overall, plastic-associated biofilms were characterized by abundant phototrophic bacteria, mainly affiliated to Cyanobacteria (up to 49.8% of total reads) of the families Leptolyngbyaceae, Nostocaceae, and Cyanobiaceae. Along with phototrophic bacteria, abundant Actinobacteria (up to 79.2% of total reads) (Supplementary Figure 5) colonized most of the analyzed samples, with the highest contribution of Propionibacteriaceae (up to 71.7% of total reads, mainly represented by the genera *Cutibacterium* and *Propionicicella*) and Atopobiaceae (up to 42.5% of total reads, mainly represented by the genus *Olsenella*). Proteobacteria were detected in all the analyzed plastisphere samples (10.4–59.6% of total reads), with most ASVs affiliated to classes Alphaproteobacteria (up to 41.9%) and Gammaproteobacteria (up to 52.9%). In particular, the genera *Sphingomonas*, *Sphingorhabdus* (family Sphingomonadaceae) and *Rhodobacter* (family Rhodobacteraceae) were mainly represented. Within Gammaproteobacteria, the most common genera (*Curvibacter*, *Burkholderia* and *Ralstonia*) belonged to the family Burkholderiaceae.

**Figure 2:**
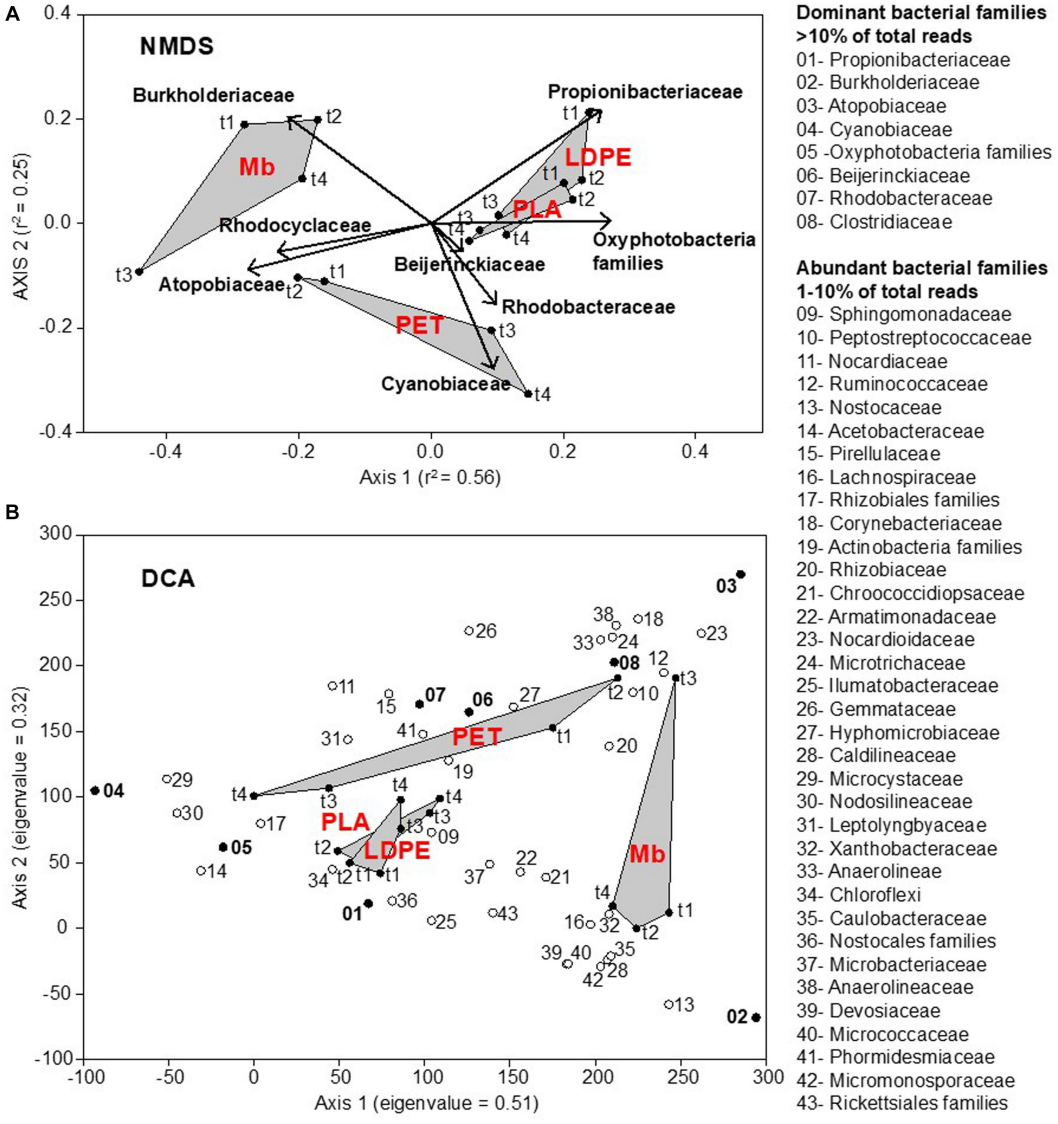
Nonmetric MultiDimensional Scaling (NMDS) ordination plot, based on Bray-Curtis (dis)similarity matrix (stress = 0.12), representing the plastisphere bacterial community composition at the family level on all plastic coupons (LDPE, PET, PLA, Mb) collected across the water immersion time (t1, t2, t3, t4). The relative abundance of dominant bacterial families (>10% of total reads: bold text) was incorporated in the analysis through a vector-fitting procedure **(A)**. Detrended Correspondence Analysis (DCA) allowing for the identification of core bacterial taxa in the plastisphere. Dominant and abundant families are numbered following their relative abundance values. Eigenvalues of ordination axes indicate their relative importance in explaining the spread in the data **(B)**.

Plastic-associated communities clustered depending on polymer type, while the changes in community composition were not consistent over time. Three distinct groups were obtained (Figure 2), one clustering biofilm communities associated with Mb, one PET communities, and one comprising both LDPE and PLA. Community composition was statistically different between polymers (PERMANOVA, *p* < 0.001), with the exception of that on LDPE and PLA (PERMANOVA, *p* > 0.001).

ASVs affiliated to Gammaproteobacteria dominated the biofilm communities grown on Mb coupons with the highest contribution from members of the family Burkholderiaceae (up to 50.5%). Actinobacteria were also present, mainly comprising Atopobiaceae (42.5%) and Propionibacteriaceae (up to 14.1%) (Supplementary Figures 2, 5).

Biofilm communities on LDPE, PET and PLA were predominantly represented by the Actinobacteria, families Propionibacteriaceae (up to 71.2%, mainly found on LDPE and PLA coupons) and Atopobiaceae (up to 38.3%, mainly found on PET), along with a relatively higher contribution of photosynthetic pigmented microorganisms in comparison to Mb (Figure 2).

Moreover, the community composition on LDPE, PET and PLA was significantly different between the initial biofilm development (t1 and t2), with the dominance of heterotrophic bacteria (Atopobiaceae and Peptostreptococcaceae on PET, Propionibacteriaceae and Sphingomonadaceae on LDPE and PLA), and the later stage of growth (t3 and t4), with the dominance of photosynthetic bacteria (Rhodobacteriaceae on PET, Cyanobiaceae and other Oxyphotobacteria families on LDPE and PLA) (PERMANOVA, *p* < 0.005).

Overall, all the analyzed plastisphere communities shared a core of ASVs affiliated to about 70 bacterial families, regardless polymer types and biofilm age (Supplementary Figure 6). Notably, 8 families were found as dominant (mainly Propionibacteriaceae, Burkholderiaceae, Atopobiaceae, Cyanobiaceae), and 34 families were abundant. All dominant (>10% of the total reads) and abundant (>1% of the total reads) bacterial taxa retrieved in the freshwater plastisphere were found in the surrounding waters, albeit showing a lower relative abundance.

### 3.3 Polymer surface analysis

Changes in polymer surface morphology, chemical composition, and functional groups were investigated by FT-IR, SEM, and contact angle analyses at the end of the experiment (day 77).

None of the polymers immersed in lake water for 77 days showed changes in surface morphology or chemical composition compared to the reference polymer items, except for the Mb sample. This suggests that biodegradation activity of the plastisphere was limited or absent.

According to our observation, DNA extraction procedure did not affect virgin plastic samples morphology nor ATR-FTIR spectra (Supplementary Figures 7, 8).

#### 3.3.1 Low density polyethylene (LDPE)

**Figure 3:**
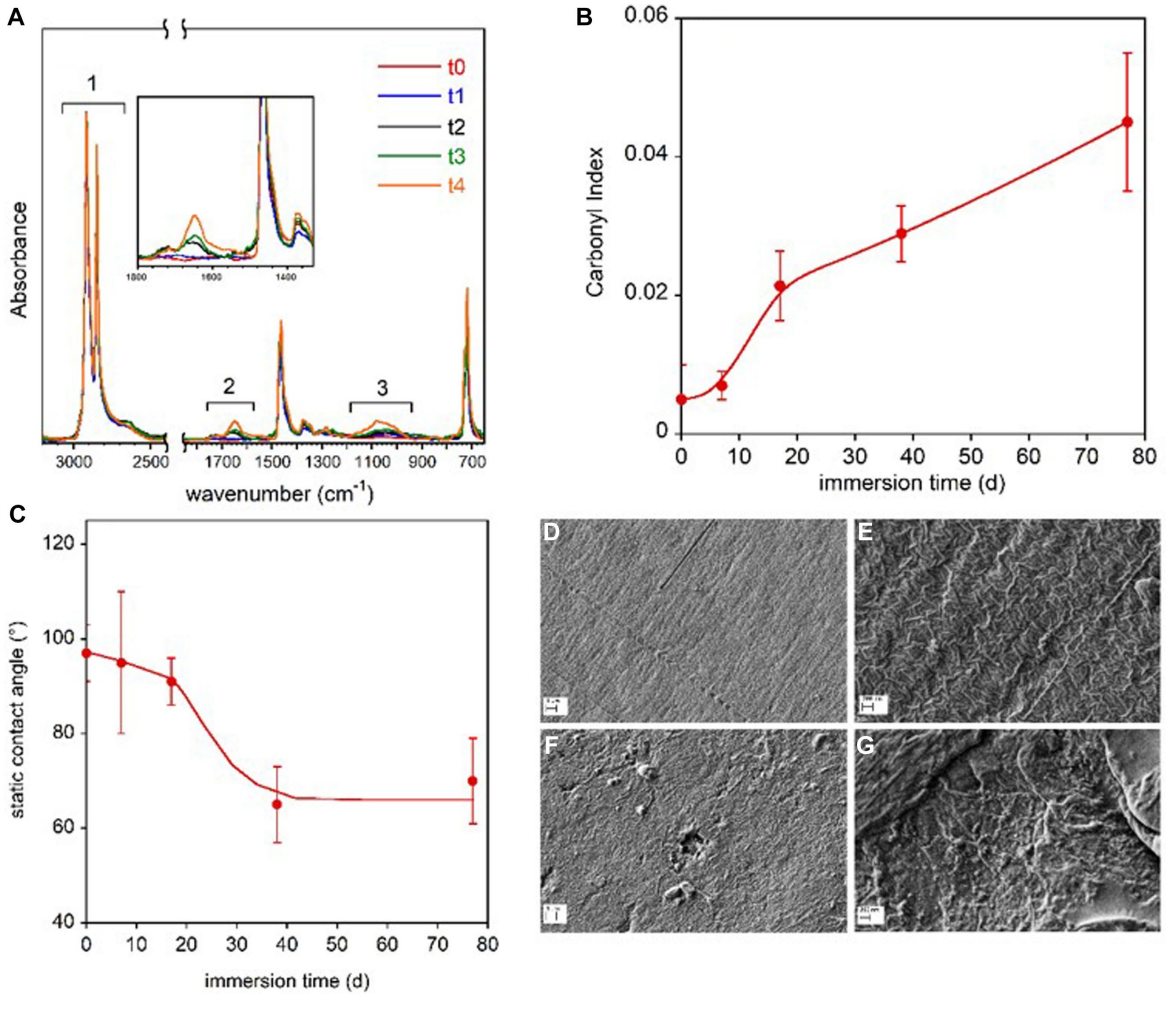
LDPE coupon surface characterization. FTIR-ATR spectra of the LDPE samples immersed in freshwater for the different time periods **(A)**. In the inset, the magnification of the spectra in the 1,800–1,350 cm^−1^ range. Carbonyl Index reported as a function of the immersion time **(B)**. Static contact angle of water on LDPE films as a function of immersion time in freshwater **(C)**. The lines in panel **(B,C)** are guides for the eyes. SEM micrographs of reference LDPE-to at two magnifications **(D,E)** and LDPE-t4 film immersed for 77 d at two magnifications **(F,G)**.

Polyethylene coupon were composed of LDPE, as shown by the typical absorption band pattern in the 1,400–1,330 cm^−1^ spectral region. The spectra of the virgin polyethylene clearly showed the presence of ink on the printed side of the coupon. Due to the heterogeneous distribution of the ink, the following analyses refer only to the non-printed surface of the film. Figure 3A shows the spectra of the samples immersed in freshwater for the different time periods. The absorbance of the spectra was normalized respect to the intensity of the band at 2,914 cm^−1^, due to the CH_2_ asymmetric stretching.

The three highlighted spectral regions are referred to the absorptions due to the symmetric and asymmetric CH_2_ stretching (1), not involved in the sample transformation, and to the C=O (2) and C-O stretching (3). In the 1,800–1,350 cm^−1^ region, the C=C stretching vibration could also resonate, but the presence of this group can be excluded because the band at 909 cm^−1^ for the vinyl moiety is absent. The progressive oxidation of the sample was evaluated by following the carbonyl index, which is the ratio of the integrated absorbance between 1,800 cm^−1^ and 1,550 cm^−1^ and between 3,000 cm^−1^ and 2,450 cm^−1^ regions, in accordance with the procedure described by Brandon et al. (2016) (Figure 3B). No marked increase in the carbonyl bands of LDPE polymer samples was observed from day 1 (t0) to day 7 (t1). Starting from day 17 (t2), the carbonyl index increased progressively until the end of the investigated period (t4, day 77). Despite the scattered values caused by the compositional and topological heterogeneity of the samples, a significant decrease in the contact angle was observed after 17 days of immersion, indicating increased wettability and higher hydrophilicity of the polymer film surface (Figure 3C).

The SEM analysis revealed surface morphology modifications. The SEM micrographs of the reference polymer coupon (t0) and of the coupon immersed for 77 d (t4) are displayed at two magnifications in Figures 3D–G. The images of the reference LDPE before the immersion (Figures 3D,E) clearly show the typical lamellar texture of a film obtained from blown extrusion (Zhang et al., 2004) while on the samples immerse in lake water for 77 days (t4), an increase of surface roughness, the presence of pits and holes as well as nano-beads and debris can be observed (Figures 3F,G).

#### 3.3.2 Polyethylene terephthalate (PET)

**Figure 4:**
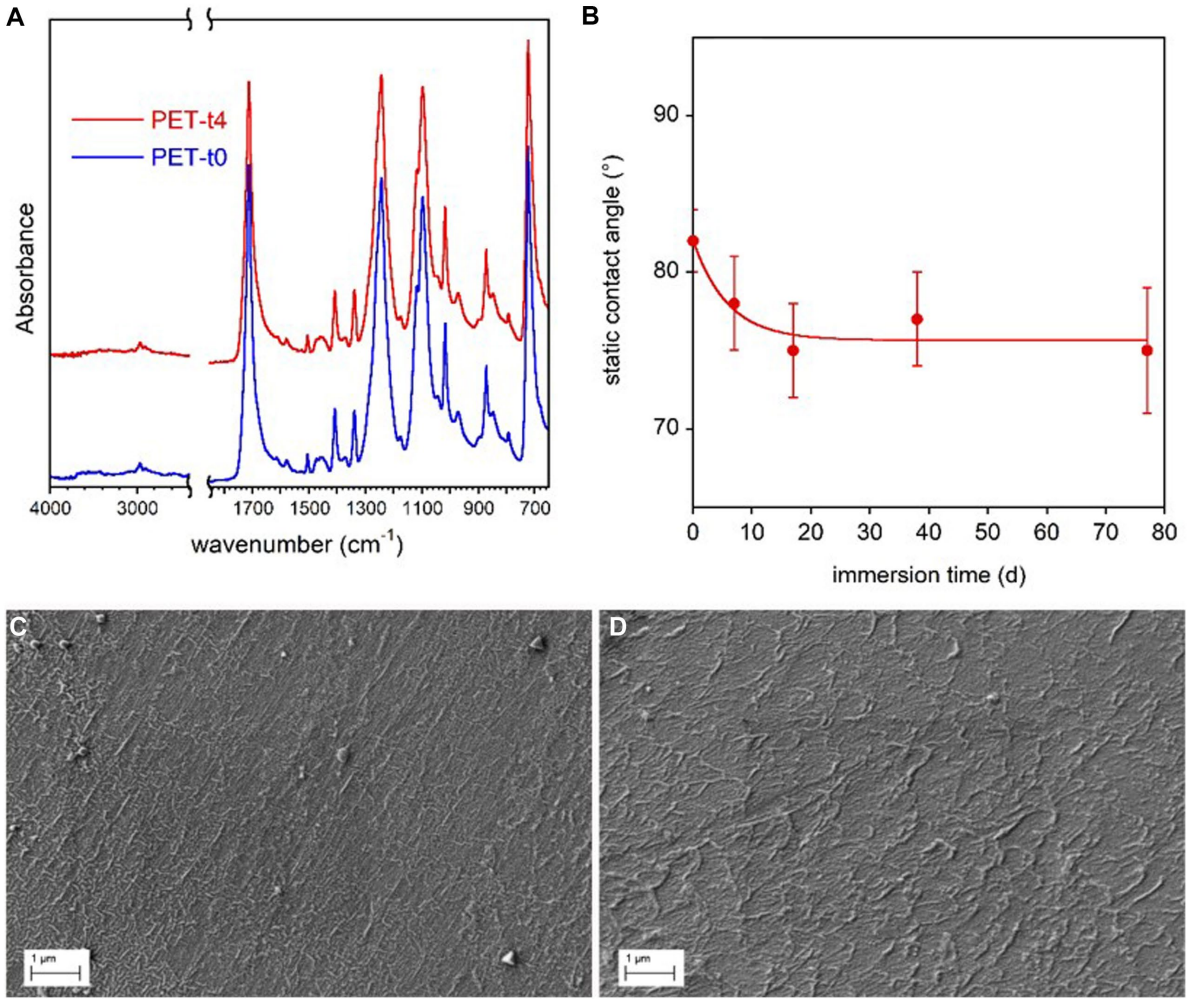
PET coupon surface characterization. FTIR-ATR spectra of the reference PET sample (t0) immersed in freshwater for 77 d (t4) **(A)**. Static contact angle of water on PET films as a function of immersion time in freshwater **(B)**. The line in panel **(B)** is guide for the eyes. SEM micrographs of reference PET at t0 **(C)** and PET immersed for 77 d (t4) **(D)**.

The FTIR spectra of all the PET samples were very similar and no new absorption bands appeared after the immersion in freshwater for different time periods (Figure 4A).

A very small increase of samples water wettability with the immersion time (Figure 4B), was observed by contact angle analysis, presumably due to the progressive removal of mold release additives used in bottle production process. In addition, SEM analysis showed no marked surface morphology variation between the reference and conditioned samples for 77 days (Figures 4C,D).

#### 3.3.3 Polylactic acid (PLA)

**Figure 5:**
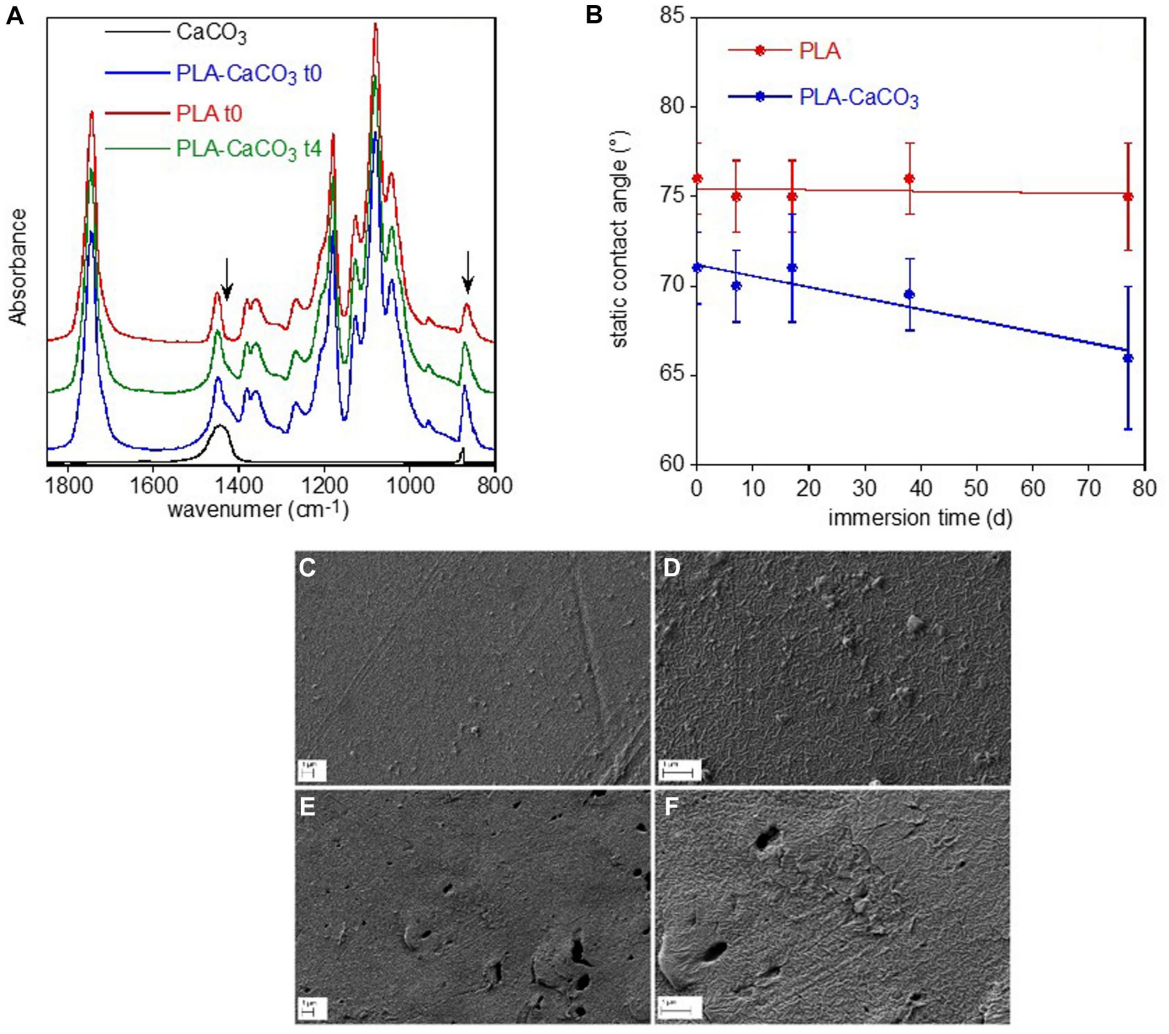
PLA sample surface characterization. FTIR-ATR spectra of the two faces of PLA reference sample, without (PLA-10) and with (PLA-CaCO_3_-10) the absorption bands of CaCO_3_ used as filler, and of the PLA-CaCO_3_- t4 sample immersed for 77 d in freshwater **(A)**. For comparison, the spectrum of CaCO_3_ was reported. The arrows indicate the spectral modifications of the conditioned sample. Static contact angle of water on the two faces PLA- CaCO3 and PLA as a function of immersion time in freshwater **(B)**. SEM micrographs of PLA-CaCO3 samples **(C–F)**. Reference PLA- CaCO3-10 sample at two different magnifications **(C,D)**, and PLA- CaCO3-14 sample immersed for 77 d at two different magnifications **(E,F)**.

**Figure 6:**
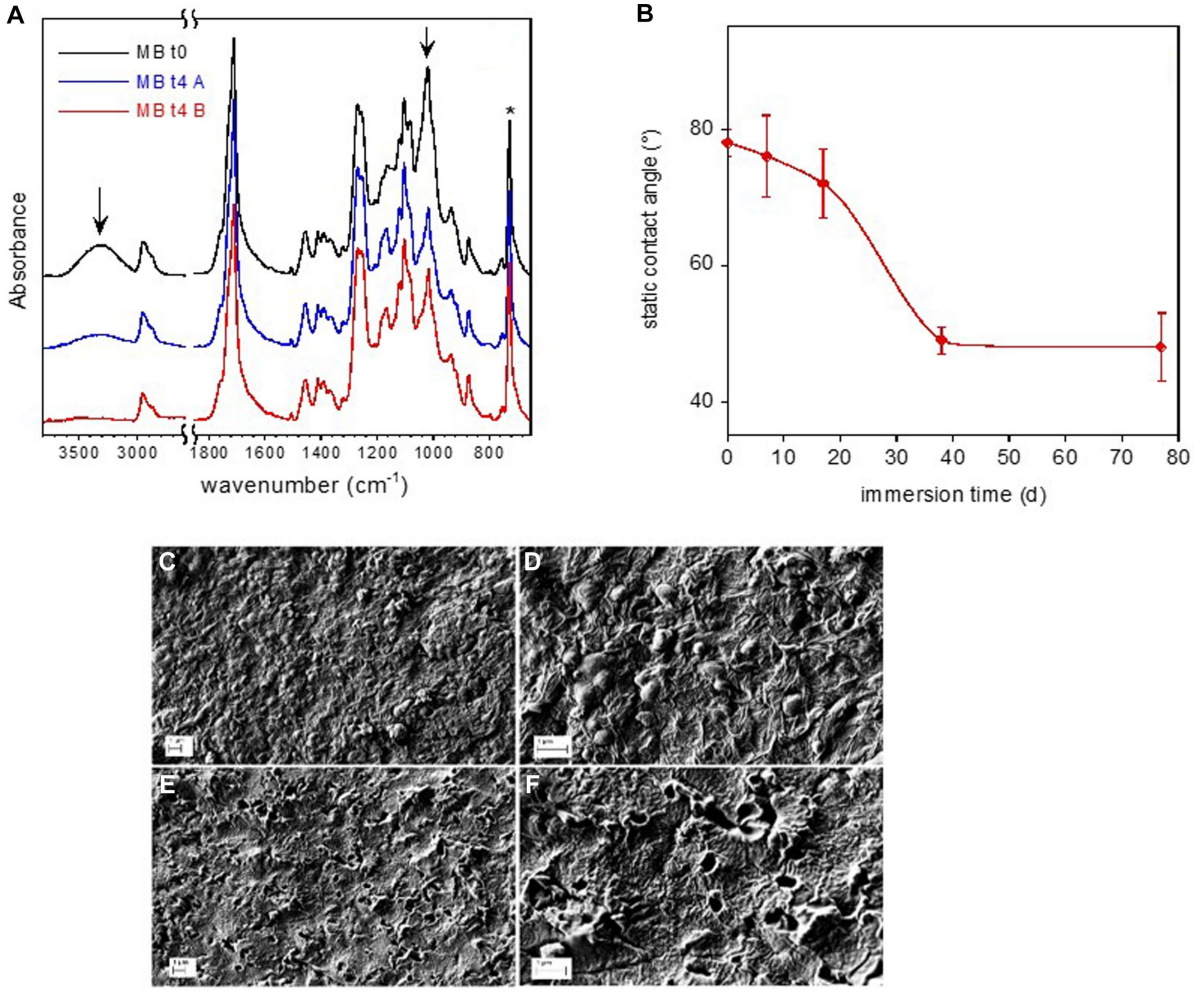
Mb coupon surface characterization. FTIR-ATR spectra of reference Mb-to and of Mb-t4 after 77 d immersion in freshwater **(A)**. The arrows indicate the absorption bands of starch. Static water contact angle on Mb film as a function of immersion time in freshwater **(B)**. SEM micrographs of Mb sample **(C–F)**. Reference Mb sample at two different magnifications **(C,D)**. Mb-t4 sample immersed for 77 d at two different magnifications **(E,F)**.

PLA sample showed different spectra on the two sides, with the bottom surface (PLA-CaCO3) presenting typical absorption bands of calcium carbonate at 1,470 cm^−1^ and 870 cm^−1^ superimposed to those of the polymer (Figure 5A). This inorganic filler is typically added to reduce the amount of the more expensive polymer and as whitening agent. The FTIR spectra did not show any variation of the polymer band intensity and position or the appearance of new bands that could be caused by biotic or abiotic hydrolysis or photo degradation via the Norrish II mechanism (Tosakul et al., 2022). The only observed spectral modification was the progressive decreases of calcium carbonate band intensity of the PLA-CaCO_3_ sample at day 77 (t4) (Figure 5A). The contact angle results show that the PLA face with the inorganic filler (PLA-CaCO_3_) was slightly more hydrophilic than the simple PLA face (Figure 5B). Moreover, the contact angle of PLA-CaCO_3_ decreased with immersion time, indicating increased wettability. This variation can be related to the increase in surface roughness, as no modification of the polymer composition has been observed. Finally, SEM showed some morphological changes in the PLA-CaCO_3_ side of the sample, as result of water immersion (Figures 5C–F). In fact, the images reported in Figures 6E,F show an increased surface roughness and the appearance of well-defined holes, likely due to the removal or partial dissolution of CaCO_3_ salt particles.

#### 3.3.4 MaterBi^®^ (Mb)

The FTIR spectra of the pristine Mb and of the sample immersed for 77 days are displayed in Figure 6A. They were normalized with respect to the band at 727 cm^−1^, assigned to the CH_2_ bending of butylene moiety (marked by an asterisk in figure). According to the FTIR analysis reported in literature, the pristine Mb coupon is composed of starch dispersed in a poly(butylene adipate-co-terephthalate) (PBAT) matrix (Weng et al., 2013; Elfehri Borchani et al., 2015). In particular, the main absorption bands of amylose and amylopectin components of starch (marked by arrows in Figure 6A) are assigned to O-H stretching at about 3,300 cm^−1^ and to the C-O stretching at 1000 cm^−1^. This last absorption is partially superimposed to that of the ester bond of the polymer.

After an immersion time of 77 days, the intensity of the bands at 3,300 cm^−1^ and 1,000 cm^−1^, assigned to the starch, decreased (Figure 6A). On the other hand, no change in band intensity of the PBAT or emergence of new absorption (e.g., due to carboxyl or carboxylate groups) can be observed, indicating no evident hydrolytic degradation of the matrix.

The surface of the Mb-t0 sample had a water contact angle of 78°, indicating moderate wettability. During the immersion time, the sample became progressively more hydrophilic, as evidenced by the contact angle decreasing to about 50° at t3 (day 38) (Figure 6B).

Immersion in freshwater also caused a major modification of the Mb film surface morphology, as shown in the SEM micrographs of the coupons at t0 (Mb-t0) and after 77 days of immersion in lake water (Mb-t4) (Figures 6C–F). The reference Mb-t0 sample clearly showed the typical texture of Mb, with starch nodules of about 200 μm in diameter embedded in a continuous PBAT matrix. After 77 days of immersion, holes were observed on the film surface at the locations of the nodules, indicating starch biodegradation within the intact PBAT matrix.

## 4 Discussion

In this study, all plastic coupons exposed to natural lake waters harbored a well-structured microbial biofilm community. A patchy distribution of microbial cells and small cell clusters, including either pigmented or non-pigmented microorganisms, was observed at the initial stage of plastisphere development, followed by a more homogeneous surface coverage due to the growth of a mature microbial biofilm filling spaces between patches.

As previously reported for stream and river biofilms (Besemer et al., 2012; Besemer, 2015), the plastisphere bacterial community was characterized by few dominant taxa and a long tail of rare taxa. The community composition of plastic-attached bacteria differed from that in surrounding waters, consistently with data available from marine (Oberbeckmann and Labrenz, 2020; Du et al., 2022; Zhang S.-J. et al., 2022) and freshwater systems (Qiang et al., 2021; Li et al., 2023; Miao et al., 2023). Most of the retrieved ASVs were affiliated to different cyanobacterial families comprising well-known freshwater biofilm-forming members (e.g., Leptolyngbyaceae, Nostocaceae, and Cyanobiaceae) (Besemer, 2015; Zancarini et al., 2017). Benthic cyanobacteria, along with pennate diatoms, colonial and filamentous green algae, were previously shown to occur in plastisphere exposed to sunlight in marine and freshwater ecosystems (Eich et al., 2015; Oberbeckmann et al., 2016; Huang et al., 2022; Zhang W. et al., 2022; Vladimir et al., 2023). Noteworthy, the majority of ASVs affiliated to Burkholderiaceae, Rhodobacteraceae, and Sphingomonadaceae in the Alphaproteobacteria and Gammaproteobacteria were found both in planktonic and plastisphere communities. Our results indicated that during the initial settlement phase, plastic microbial colonizers and pioneering opportunistic microorganisms were likely recruited from the free living planktonic community. Members of these recruited families are commonly known to produce extracellular polymeric substances to facilitate cell adhesion and secondary colonizers.

In spite of the similar diversities value over time regardless on the polymer type, significant differences in taxon composition were found between communities grown on different plastic polymers. Interestingly, no polymer-specific taxa were observed and a common core microbiome was found in all plastisphere. The dominant core taxa were likely to shape plastic communities rather than rare ones.

Noteworthy, the starch-based Mb showed consistent differences of microbial composition and surface modification in respect to the other analyzed polymers.

Regarding plastisphere composition, LDPE, PET and PLA selected for members of Actinobacteria (up to 79.2%), during the initial phases of development (t1 and t2), and phototrophic bacteria at later stages of biofilm growth (t3 and t4). On the contrary, Mb coupons biofilm was mainly enriched of Gammaproteobacteria members of the family Burkholderiaceae (contributing up to 50.5% of the total reads).

Similarly, plastic surface analysis revealed no obvious polymer modifications due to the biofilm colonization in LDPE, PET and PLA while it showed some evidence of biodegradation on the Mb samples. Both the ATR-FTIR and SEM analyses showed the selective degradation of starch granules of the Mb sample surface, suggesting biodegradation from the biofilm microorganisms.

The composition pattern of biofilms developed on the starch-based Mb substrata, particularly at the initial stages of biofilm growth, may be driven by microorganisms suitable to compete for polymer carbon as a growth substrate. In this regard, the dominance of family Burkholderiaceae in Mb may be explained by their capacity to degrade complex carbon compounds (Besemer, 2016; Zancarini et al., 2017), as the starch present in granules in the polymer structure. Despite starch granule degradation, the PBAT matrix maintained its original chemical structure and morphology, indicating limited biodegradability of Mb following the prolonged exposure to lake waters. It has been reported that erosion of Mb in simulated abiotic seawater for 45 days, in addition to extensive starch leaching, resulted in a profound morphological change presumably due to PBAT degradation (Denaro et al., 2020). In this study, large drop in contact angle values was instead mainly due to the progressive surface roughness increase and the formation of holes due to starch nodules disappearance. ATR-FTIR did not show any variation of the PBAT matrix composition which, otherwise, could have favor a higher hydrophilicity.

No polymer modifications due to biotic activity were observed on LDPE and PET and, unexpectedly, also on the biodegradable PLA sample surfaces. The surface of PET and PLA were indeed not subjected to polymer chemical modification but only to inorganic filler (CaCO_3_) dissolution. Differently, a clear evolution of LDPE film properties, such as an increase of the oxidation degree, hydrophilicity and surface roughness, was observed. A similar behavior was reported in biotic or abiotic conditions (Zadjelovic et al., 2022). However, it was also reported that in biotic environment the oxidized groups on PE surfaces decreased because of their consumption by microorganism consortia (Hasan et al., 2007; Kumar Sen and Raut, 2015; Novotný et al., 2018) and that the increase of the absorption band related to the double bond formation occurred in samples aged under natural conditions (Hakkarainen and Albertsson, 2004). According to these previous observations our results clearly suggested that the observed LDPE oxidation was likely originated from abiotic degradation processes (e.g., sunlight irradiation).

Complex communities may be more efficient degraders of plastic polymers than single microbial strains for their higher metabolic versatility (Jacquin et al., 2019). However, the absence of a clear biodegradation in our study suggested that complex biofilm communities, grown after 77 days of immersion on LDPE, PET and PLA, did not enhanced the metabolic capacity to hydrolyse and use the plastic polymers as carbon source.

Thus, we concluded that microorganisms associated with LDPE, PET and PLA, mostly used polymers as adhesion surfaces. This is consistent with the few previous studies evaluating the plastic biodegrading capacity of marine plastisphere (Oberbeckmann et al., 2021; De Monte et al., 2022; Wallbank et al., 2022). The limited surface modification on LDPE, PET and PLA, observed in our study, might be also due to the protective action of the biofilm colonies, especially in samples with abundant photosynthetic microorganisms. The presence of Rhodobacteriaceae on PET, Cyanobiaceae on LDPE and PLA, along with microalgae and diatoms in all the polymers tested, might provide a further attachment substratum for bacteria. Biofilm-forming cyanobacteria and diatoms are known to highly contribute to EPS secretion (Duan et al., 2023; Raghavan et al., 2023; Romeu et al., 2023; Zackova Suchanova et al., 2023), decreasing the chance for the bacterial populations, including those potentially suitable to degrade polymers, to be in direct contact with plastic surfaces. Moreover, Cyanobacteria and microalgae provide assimilable organic matter (e.g., photosynthates, EPS) (Underwood and Paterson, 2003), that is more likely to be used as growth substrate than carbon of plastic materials.

Overall, our results showed limited or no degradation of plastic polymers independently on their expected biodegradability (i.e., PLA and Mb), suggesting that laboratory tests (Tabasi and Ajji, 2015) might be of limited value to describe plastic polymer fate in surface water.

The different taxon composition retrieved between the microbiome of the PET communities and those of LDPE and PLA in the initial stage of biofilm development might be explained by the diverse polymer surface tension which affects the adherence of bacterial cells. In fact, the pairwise favorable and unfavorable interaction between the bacterial cells and the substratum is considered to be a key factor governed by the respective surface tensions (Fletcher and Pringle, 1983; Mceldowney and Fletcher, 1986; Liu and Zhao, 2005; Krasowska and Sigler, 2014). Interestingly, LDPE and PLA polymers, especially at the initial stage of development, showed similar surface tension, respectively 30 mN m^−1^ (Jańczuk and Białopiotrowicz, 1990) and 30–37 mN m^−1^ (Jordá-Vilaplana et al., 2014), lower than those of PET (41 mN m^−1^) (Jańczuk and Białopiotrowicz, 1990).

## 5 Conclusion

In this study, we found that plastic coupons exposed to natural lake waters developed a diverse microbial biofilm community, with initial patchy distribution followed by a more homogeneous coverage of the surface. The composition of plastic-attached microbial community differed from the surrounding water, with a higher contribution of photosynthetic pigmented cells. Although further replicates from each polymer are likely necessary at longer incubation times, our results suggested that the polymer type could affect plastisphere composition regardless the biofilm age.

The study outcomes indicated that different plastic materials, either derived from petrochemical hydrocarbons (i.e., LDPE and PET) or biodegradable (PLA and Mb), are mostly used by opportunistic aquatic microorganisms as adhesion surfaces rather than carbon source. Mb coupons promoted the occurrence of Gammaproteobacteria members of the family Burkholderiaceae, which were likely to degrade the starch granules within the polymer structure, but without any consistent chemical and morphological effect on the PBAT matrix. Overall, our findings emphasize the necessity to further address the impact of extended incubation periods at varying environmental conditions on the potential biodegradation of plastic debris in the aquatic environment.

## Data availability statement

The data presented in the study are deposited in the Sequence Read Archive (SRA) repository, accession number PRJNA905066.

## Author contributions

FP: Conceptualization, Data curation, Methodology, Supervision, Writing – original draft, Writing – review & editing. VB: Data curation, Formal analysis, Investigation, Methodology, Writing – original draft. SA: Data curation, Formal analysis, Methodology, Writing – review & editing. SC: Formal analysis, Methodology, Writing – review & editing. CL: Data curation, Funding acquisition, Writing – review & editing. LP: Conceptualization, Methodology, Writing – review & editing. VL: Writing – review & editing, Methodology. AM: Conceptualization, Investigation, Writing – review & editing, Supervision. SR: Conceptualization, Investigation, Writing – review & editing.

## Funding

The author(s) declare that no financial support was received for the research, authorship, and/or publication of this article.

## Footnotes

^1^ https://github.com/qiime2/q2-demux

^2^ https://github.com/qiime2/q2-cutadapt
