# Supplementary material for: Microbial colonization patterns and biodegradation of petrochemical and biodegradable plastics in lake waters: insights from a field experiment
Source: Front Microbiol. 2023 Dec 6;14:1290441. doi: 10.3389/fmicb.2023.1290441 (PMC10731271; doi:10.3389/fmicb.2023.1290441)
Supplement: Supplementary file 1 [file Data_Sheet_1.PDF]

Microbial colonization patterns and biodegradation of petrochemical and biodegradable plastics in lake waters: insights from a field experiment

Francesca Di Pippo[1*], Valerio Bocci[1,2], Stefano Amalfitano[1,3], Simona Crognale[1,3], Caterina Levantesi[1], Loris Pietrelli[4], Valerio Di Lisio[5], Andrea Martinelli[6], Simona Rossetti[1]

[1]Water Research Institute, CNR-IRSA, National Research Council, Rome, Italy

[2] PhD program in Evolutionary Biology and Ecology, Dept. of Biology, University of Rome 'Tor Vergata', Rome, Italy

[3] National Biodiversity Future Center, Palermo, Italy

[4] DAFNE Department, Tuscia University, Viterbo, Italy

[5] Donostia International Physics Center, Paseo Manuel de Lardizabal, San Sebastian, Spain

[6] Department of Chemistry, Sapienza University of Rome, Rome, Italy

Supplementary Material

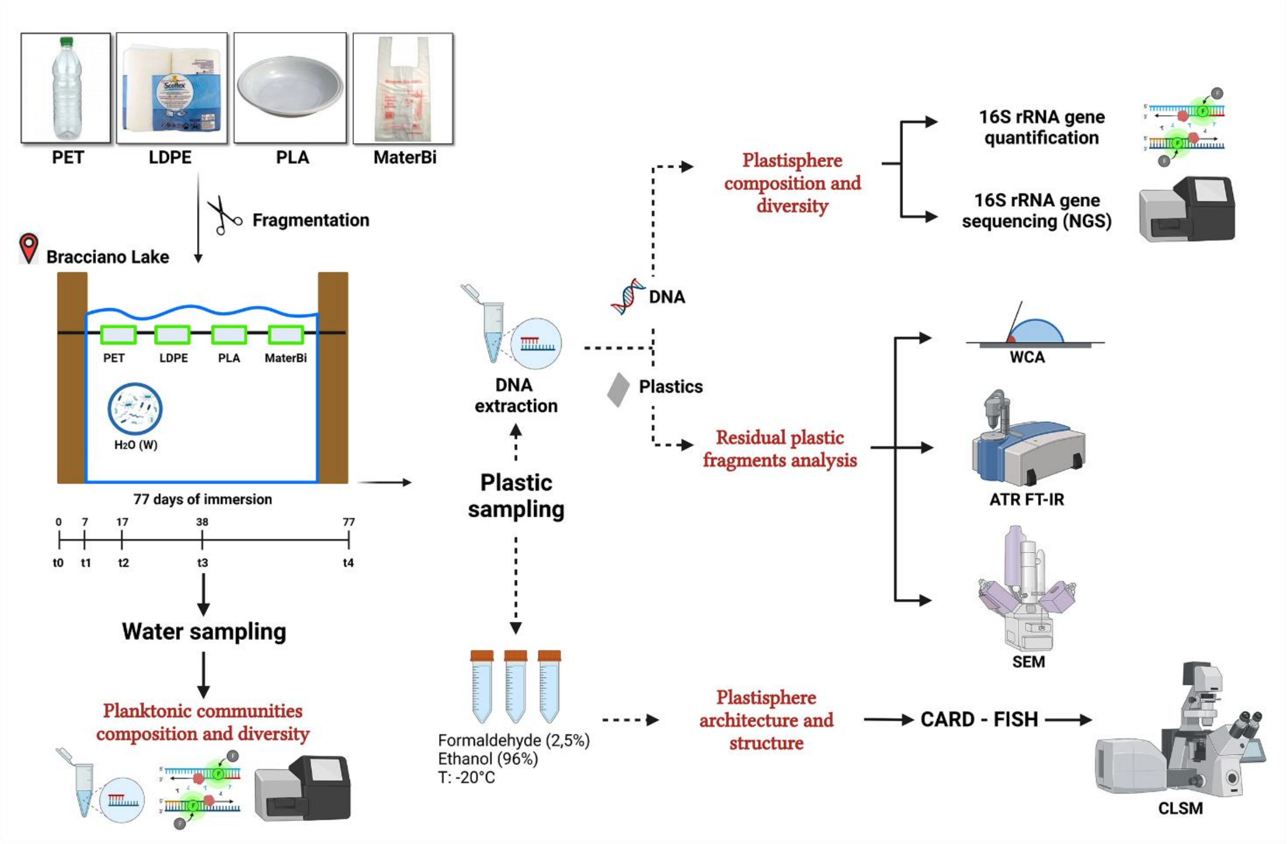

Supplementary Figure 1. Experimental set up used in this study (images were created using BioRender.com).

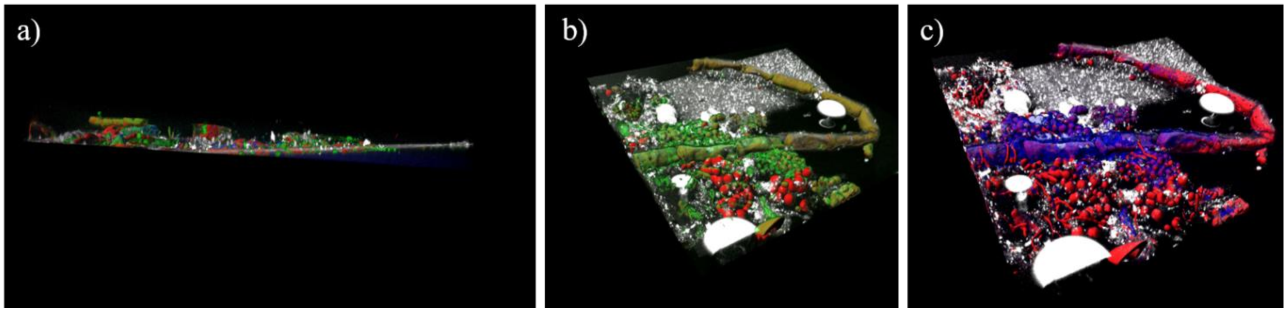

Supplementary Figure 2. 3-D reconstruction of a biofilm communities from PET (a) and Mb (b, c) after 77 days of exposure to lake waters.

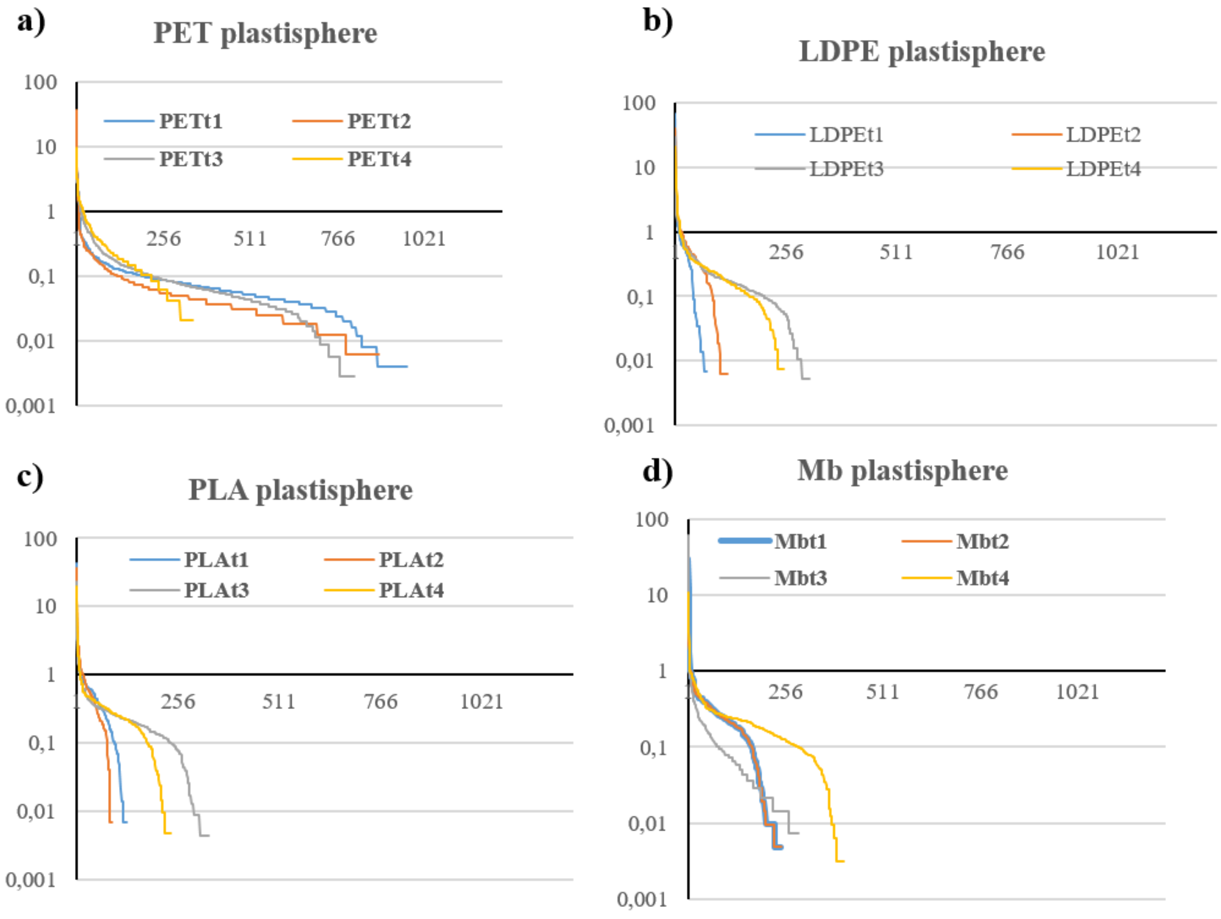

Supplementary Figure 3.: Rank–abundance curves of plastisphere bacterial communities. Curves are displayed on a log–log scale for clarity.

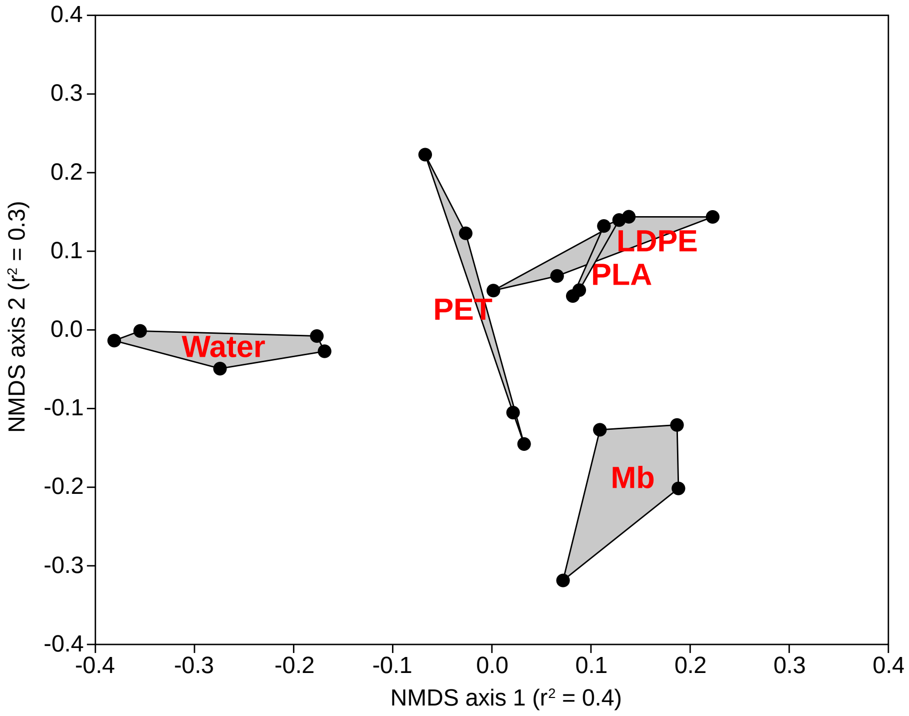

Supplementary Figure 4. Nonmetric MultiDimensional Scaling (NMDS) ordination plot, based on Bray-Curtis (dis)similarity matrix (stress = 0.16), representing the bacterial community composition at the family level in water samples and on plastic coupons (LDPE, PET, PLA, Mb) collected across the experiment.

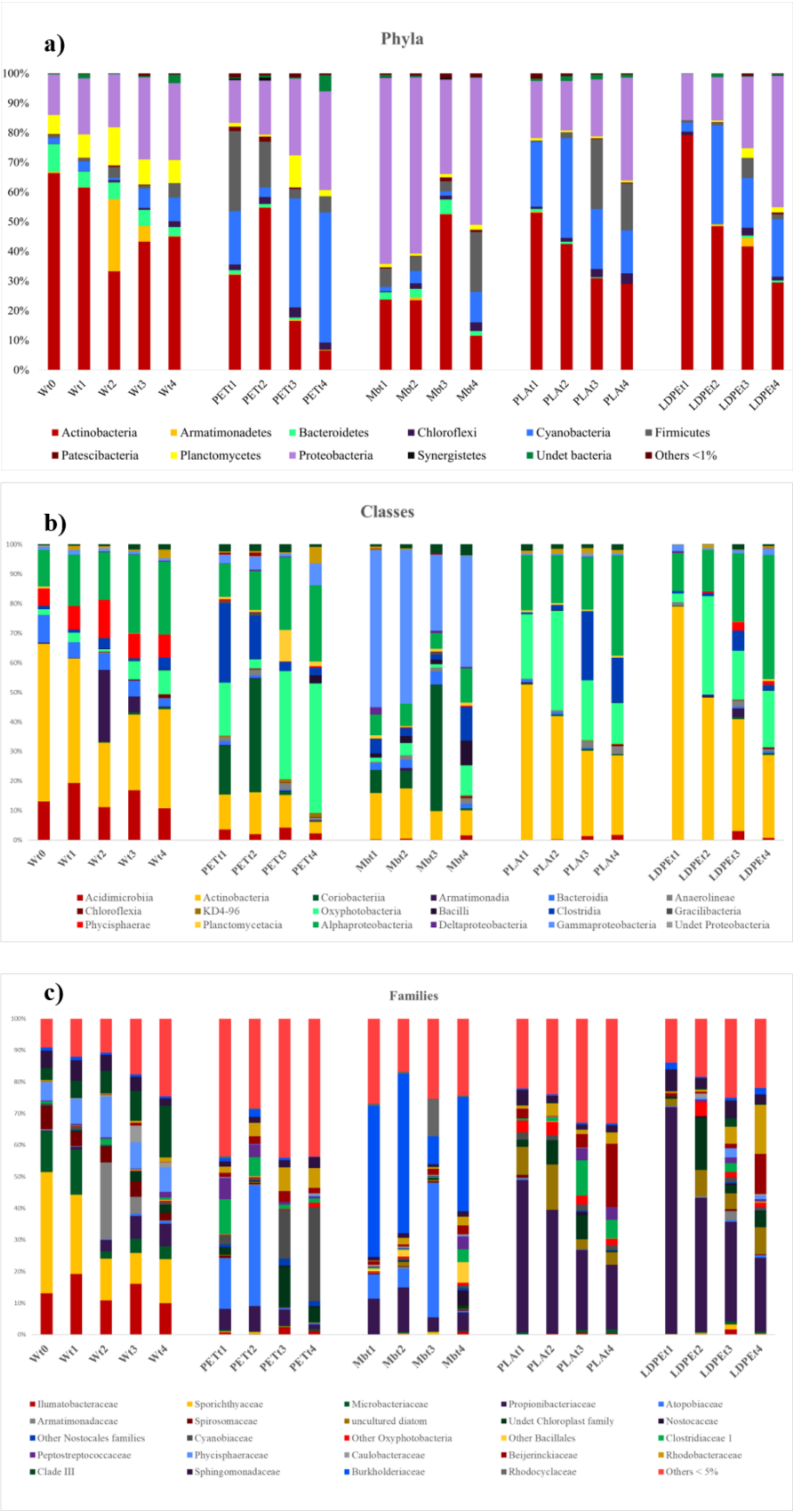

Supplementary Figure 5. Relative abundance (%) of most representative phyla (a), classes (b) and families (c) in planktonic communities and plastic-associated biofilms grown on different polymer coupon surfaces over the experimental time. Data are reported as percentage of total reads

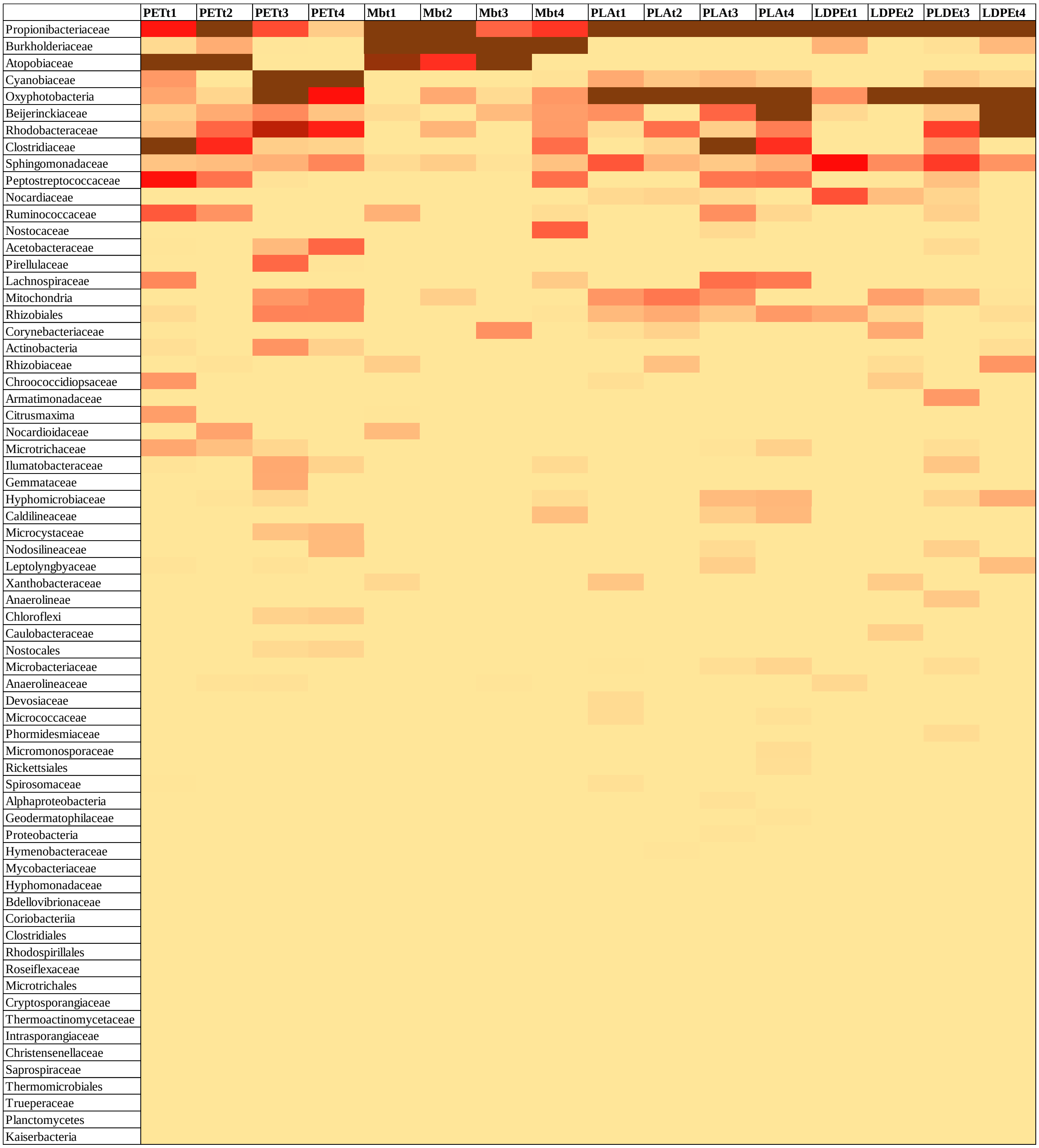

Supplementary Figure 6. Heat map of the core microbiome (families) shared by the plastic-attached biofilms

Additional test on virgin plastic samples

To exclude possible effects of DNA extraction procedure on morphology and composition of polymer coupons, additional tests were performed by SEM and ATR-FTIR on virgin polymer samples after DNA extraction procedure.

Virgin plastic samples after the DNA extraction procedure showed no morphological variation of the surface compared to pristine coupons, as evidenced by SEM images in Supplementary Figure 7.

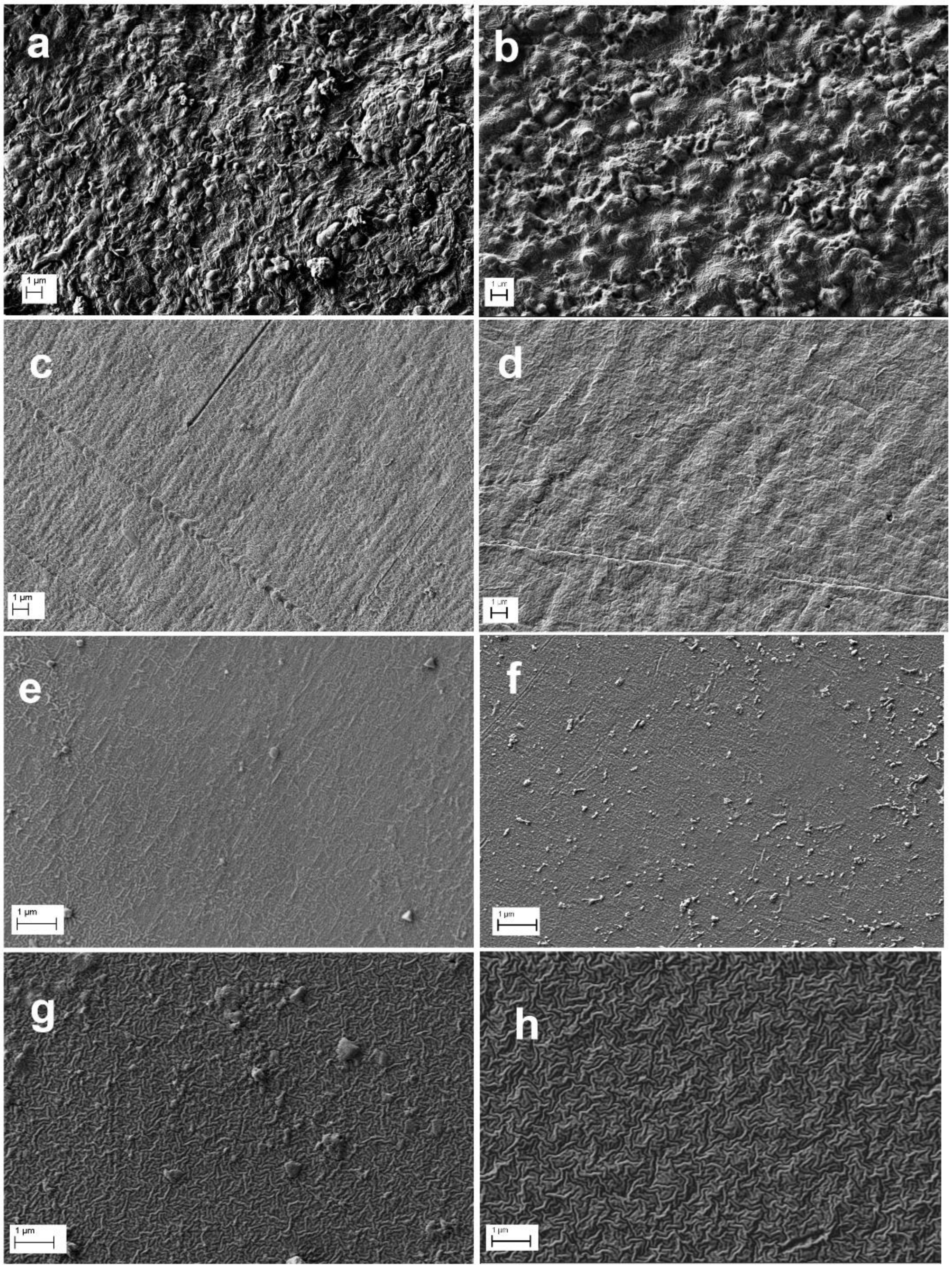

Supplementary Figure 7. SEM images of virgin plastic samples before (a, c, e, g) and after (b, d, f, h) the DNA extraction procedure. Mb (a, b), LDPE (c, d), PET (e, f) and PLA (g, h).

Virgin plastic samples after the DNA extraction procedure showed no ATR-FTIR spectra changes compared to pristine coupons, as evidenced in Supplementary Figure 8.

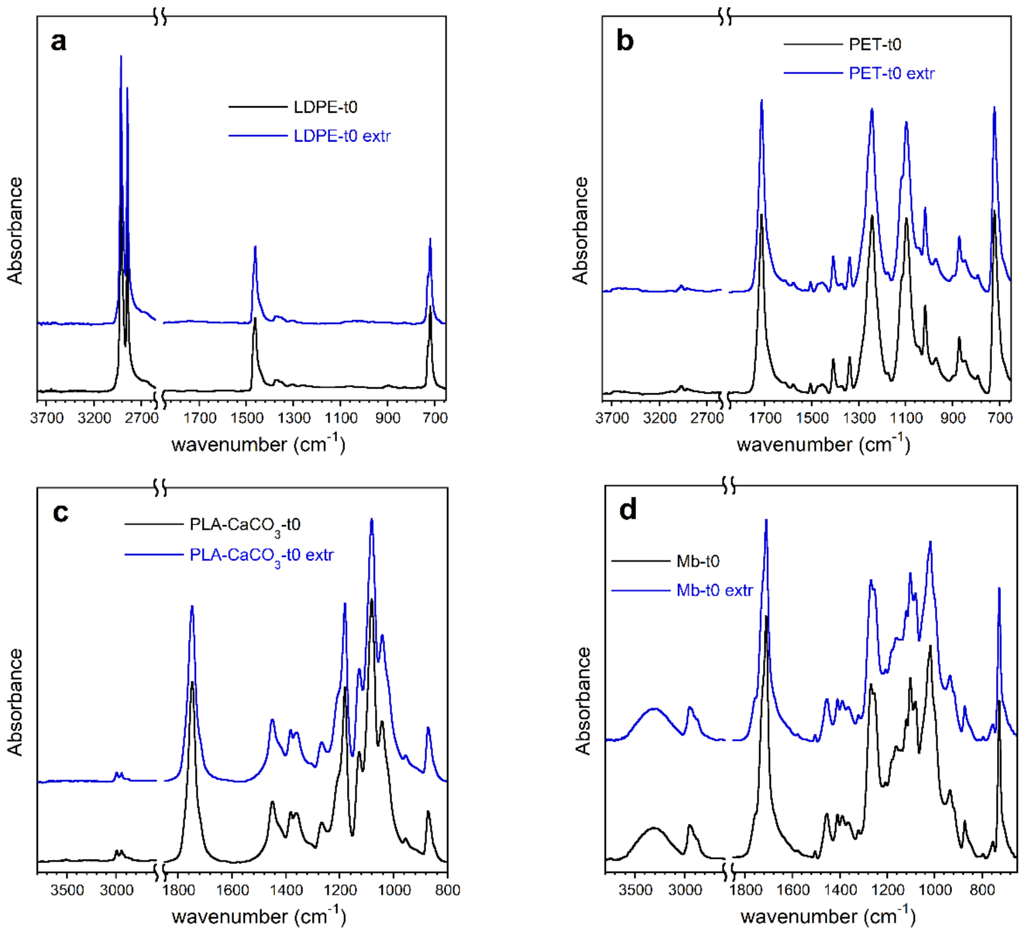

Supplementary Figure 8. ATR-FTIR spectra of virgin plastic sample before (LDPE-t0, a; PET-t0, b; PLA-$CaCO_3$-t0, c; Mb-t0, d) and after (LDPE-t0 extr, a; PET-t0 extr, b; PLA-$CaCO_3$-t0 extr, c; Mb-t0 extr, d) the DNA extraction procedure.

Supplementary Table1. 16S rDNA gene quantification (GC cm^{-2}).

	Days			
	**7**	**17**	**38**	**77**
**PET**	$6.43x10^6±5.83x10^6$	$1.11x10^7±5.47x10^6$	$1.24x10^7±4.90x10^6$	$1.05x10^7±1.85x10^6$
**LDPE**	$6.78x10^6±8.74x10^6$	$3.17x10^7±1.33x10^7$	$3.26x10^7±2.24x10^7$	$6.30x10^7±3.06x10^7$
**PLA**	$1.60x10^5±3.73x10^4$	$1.94x10^7±2.64x10^7$	$4.11x10^7±1.75x10^7$	$3.87x10^7±1.23x10^7$
**Mb**	$1.10x10^6±2.47x10^6$	$7.28x10^4±2.55x10^4$	$1.72x10^6±8.66x10^5$	$3.08x10^5±2.66x10^5$

Standard deviation values are based on three experimental replicates

Supplementary Table2. Shannon index values

	Days			
	**7**	**17**	**38**	**77**
**PET**	4.87	6.04	5.67	10.42
**LDPE**	3.45	6.30	3.43	1.75
**PLA**	5.80	5.94	5.04	3.78
**Mb**	2.53	3.57	3.40	5.04
